# Diversity and Antioxidant Activity of Culturable Endophytic Fungi from Alpine Plants of *Rhodiola crenulata*, *R. angusta*, and *R. sachalinensis*

**DOI:** 10.1371/journal.pone.0118204

**Published:** 2015-03-13

**Authors:** Jin-Long Cui, Ting-Ting Guo, Zhen-Xing Ren, Na-Sha Zhang, Meng-Liang Wang

**Affiliations:** 1 Institute of Applied Chemistry, Shanxi University, Taiyuan, Shanxi Province, People’s Republic of China; 2 Institute of Biotechnology, Shanxi University, Taiyuan, Shanxi Province, People’s Republic of China; Graz University of Technology (TU Graz), AUSTRIA

## Abstract

*Rhodiola* spp. are rare and endangered alpine plants widely used as medicines and food additives by many civilizations since ancient times. Their main effective ingredients (such as salidroside and *p*-tyrosol) are praised to exhibit pharmacologic effects on high-altitude sickness and possess anti-aging and other adaptogenic capacities based on their antioxidant properties. In this study, 347 endophytic fungi were isolated from *R. crenulata*, *R. angusta*, and *R. sachalinensis*, and the molecular diversity and antioxidant activities of these fungi were investigated for the first time. These fungi were categorized into 180 morphotypes based on cultural characteristics, and their rRNA gene ITS sequences were analyzed by BLAST search in the GenBank database. Except for 12 unidentified fungi (6.67%), all others were affiliated to at least 57 genera in 20 orders of four phyla, namely, Ascomycota (88.89%), Basidiomycota (2.78%), Zygomycota (1.11%), and Glomeromycota (0.56%), which exhibited high abundance and diversity. Antioxidant assay showed that the DPPH radical-scavenging rates of 114 isolates (63.33%) were >50%, and those of five isolates (Rct45, Rct63, Rct64, Rac76, and Rsc57) were >90%. The EC50 values of five antioxidant assays suggested significant potential of these fungi on scavenging DPPH•, O_2_−•, and OH• radicals, as well as scavenging nitrite and chelating Fe^2+^, which showed preference and selection between endophytic fungi and their hosts. Further research also provided the first evidence that Rac12 could produce salidrosides and p-tyrosol. Results suggested that versatile endophytic fungi associated with Rhodiola known as antioxidants could be exploited as potential sources of novel antioxidant products.

## Introduction

**Fig 1:**
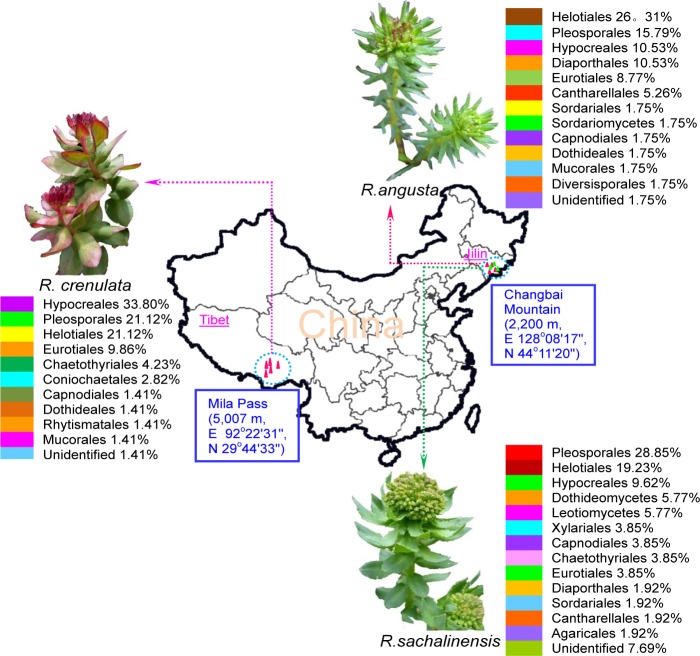
Location of three *Rhodiola* plants and distributions of endophytic fungi in their hosts. Endophytic fungi were affiliated to at least 11, 13, and 14 orders in *R*. *crenulata*, *R*. *angusta*, and *R*. *sachalinensis*, respectively, which exhibited high diversity.

*Rhodiola rosea*, also known as rhodiola, rosenroot, orpin rose, roseroot, golden root, or arctic root [1], is a perennial herbaceous plant that belongs to the family Crassulaceae and genus *Rhodiola* [2]. This species is mainly distributed in high altitudes of >2,000 m in the Arctic and mountainous regions throughout Asia and Europe [2]. This typical alpine plant has been widely used as an important food crop and folk medicine since ancient times by many countries, such as Sweden, Russia, India, and China [3,4]. More than 90 various species of *Rhodiola* have been identified, and at least 70 species are found in China [4]. Those Chinese species are mainly located in two radiated regions between the Qinghai–Tibet Plateau [5] and Changbai mountain in northeast China [6], which are about 4000 km away (Fig. 1). In China, three species, namely, *Rhodiola crenulata* (*Rc*), *Rhodiola angusta* (*Ra*), and *Rhodiola sachalinensis* (*Rs*), are significant in marketing and planting. Among these species, *Rc* is native to the Qinghai–Tibet Plateau and the only original plant according to the “Pharmacopeia of China” [7]. *Rs* and *Ra* are representative species in northeast China and comprise the largest planting area; these two species are most widely used for dietary supplements and healthy medicine.

*Rhodiola* rhizome, as a traditional folk medicine, stimulates mental and physical endurance, counteracts depression, improves sleep quality, and prevents high-altitude sickness [8,9]. Modern pharmacology research suggests that *Rhodiola* rhizome has received considerable attention because of its biological behavior, including antioxidant [10] and anti-aging properties [11], anti-microwave radiation [12], anti-hypoxia [13], and adaptogenic activities [8]. Most of these effects are ascribed to phenolics, such as salidrosides, *p*-tyrosol, and glycosides like rosavins [14], and their fundamental property is antioxidant activity [15]. Several *Rhodiola*-rhizome-based healthy foods and medicines possess high antioxidant capacities [1].

Endophytic fungi inhabit asymptomatically and internally within various tissues of host plants during at least one stage of their life cycle [16]. These fungi are found in almost all plants [17]. Although many endophytic fungi have been described and explored from various terrestrial plants [17,18], only a limited number have been studied, compared with approximately one million species worldwide [19]. Other plants, particularly those with medical significance tenaciously living under extreme conditions, such as in high-altitude mountainous, oceanic, polar, and glacier areas, may harbor unique and diverse endophytic fungi [20]. However, such fungi have been rarely studied.

Since the discovery of paclitaxel, which is a powerful anticancer agent from endophytic fungi, such as *Taxomyces andreanae* and *Pestalotia* spp. [21,22], endophytes as novel sources of bioactive metabolites including anticancer, antimicrobial, anti-malarial, and other activities have elicited much attention from researchers worldwide [23]. Consequently, scientists speculate that several horizontal gene transfers occur between endophytes and their host plants [24] and jointly cope with biotic and abiotic stresses [25], which imply that preference and selectivity exist between host plants and their fungal partners [26]. Although many aspects of biology and interrelatedness of endophytes with their respective hosts have been vastly investigated [27], novel specific bioactive products and endophytes should be explored from those related pharmaceutical host plants with long history of folk use [17,21,22,27,28], which could be remarkably helpful in directing the search for bioactive products [27].

Reactive oxygen species (ROS), such as O_2_^−^, H_2_O_2_, and OH, are generated byproducts of biological reactions, which can cause oxidative damage to biomolecules and play vital roles in programmed cell death and stress-response signaling in conjunction with antioxidant production [29]. ROS and antioxidants, as both universal and evolutionarily conserved, are likely to play important role(s) in symbiotic interactions [30,31]. Asymptomatic fungi as mediators can produce antioxidants that can interrupt the chain reaction of ROS to help host plants respond to various biotic and abiotic stresses [31,32]. As a result, some endophytic fungi with scavenging ROS activity *in vitro* are isolated from special antioxidant plants [33]. However, similar studies are very few [29,34]. To date, *Rhodiola* plants with excellent antioxidant capacities have not yet been reported. The present study aimed to provide the first evidence of the diversity of culturable endophytic fungi within the *Rc*, *Ra*, and *Rs* rhizomes and investigate endophytic fungi with antioxidant activities to explore the potential sources of novel natural antioxidants.

## Materials and Methods

### Ethics Statement

The wild plants used in this study are locally protected species in China. Permission was obtained from the management committees of the Tibet Institute of Plateau Biology and the Changbai Mountain National Nature Reserve prior to collection.

### Sample Collection and Isolation of Endophytic Fungi

Rhizomes of *Rc* (*R*. *crenulata*) were collected from Mila Pass (altitude 5,007 m, E 92°22′31″–92°24′88″, N 29°44′33″–29°46′56″), Tibet Autonomous Region. Rhizomes of *Ra* (*R*. *angusta*) and *Rs* (*R*. *sachalinensis*) were collected from Changbai Mountain (altitude 2,200 m, E 128°08′17″–128°11′10″, N 44°11′20″–44°13′29″), Jilin Province, China in 2012 (Fig. 1). A total of 200 rhizome segments were selected for fungal isolations in each *Rhodiola* species (Isolation ration = number of fragments colonized by fungi / total number of fragments examined). Fresh rhizomes of *Rhodiola* spp. were thoroughly washed three times with sterile distilled water, surface sterilized with 75% ethanol for 1 min, 5% NaOCl for 5 min, and sterile distilled water twice [35]. Each sample was then cut into 2 mm to 3 mm sections and placed on potato dextrose agar at 25°C without light. Voucher specimens of plant and fungal culture were recorded and deposited in the Microbe Preservation Center of Biochemical Engineering, Shanxi University. When the purified fungi grew up to 5 cm to 8 cm in diameter, their colony characteristics (growth rate, shape, edge, color, texture, elevation, transparency, etc.) were examined.

### DNA Extraction, PCR Amplification, Sequencing, and Molecular Identification

Genomic DNA was extracted from pure mycelia using E.Z.N.A. Fungal DNA Mini Kit (Omega Bio-Tek, Doraville, GA, USA). PCR amplification of ITS rRNA gene regions followed the method of Cui et al. [35] using primer pairs ITS1 and ITS4. The PCR reaction system (25 μL total volume) contained 1 μL of template, 1 μL of each primer (5 μM each), 12.5μL of Taq PCR mix (TransGen, Beijing), and 9.5 μL of ddH_2_O. The PCR reaction was performed with the following cycles: (1) 94°C for 3 min; (2) 30 cycles of 94°C for 30 s, 55°C for 30 s, and 72°C for 1 min; and (3) 72°C for 10 min. The amplified products were purified and sequenced by Shanghai Sangon Company (Shanghai, China). The sequences were then blasted against the NCBI database (http://www.ncbi.nlm.nih.gov) of known isolates. Only those matches of sequences with high similarity published in previous studies were used. All the identified isolates were categorized into level of species, genus, or family according to ownership criterion: same species with the sequence similarity (SS) ≥ 99%, two same genera with SS ≥ 95%, and same families with SS < 95% [36]. Sequence data were submitted and deposited in GenBank under accession numbers KJ542191–KJ542358 (S1 Table).

### Preparation of Endophytic Fungal Extract

Each fungus was inoculated into a 500 mL Erlenmeyer flask containing 100 mL of liquid Czapek–Dox medium: NaNO_3_, 3 g; K_2_HPO_3_, 1 g; MgSO_4_ 7H_2_O, 0.5 g; KCl, 0.5 g; FeSO_4_, 0.01 g; sucrose, 30 g; and deionized water, 1000 mL. All shake flasks were incubated at 180 rpm on a rotary shaker at 25°C for 10 d. Each fungal broth was separated into mycelia mat and culture filtrate by Whatman No. 1 filter paper. The mycelia mat was dried and then extracted twice with 75% ethyl alcohol by ultrasonic condition. Subsequently, the filtrate was evaporated under reduced pressure (8 × 10^3^ Pa) to yield an extract and then extracted twice with 75% ethyl alcohol.

### Experimental Design of Antioxidant Activity Assay

A total of 180 extracts of representative isolates at a concentration of 10 mg mL^-1^ were examined by 1,1-diphenyl-2-picryl-hydrazyl (DPPH) free radical-scavenging assay to confirm the antioxidant activity. Extracts of isolates with high antioxidant activities at different concentrations (1 mg mL^-1^–10 mg mL^-1^) were further assayed through DPPH, superoxide anion, and hydroxyl radical-scavenging, nitrite-scavenging, and ferrous ion-chelating activity assays. The EC_50_ (effective concentration at which ROS radicals or ions were scavenged by 50%) was obtained by interpolation from linear regression analysis.

DPPH radical-scavenging activities were performed as described by Zhao et al. [33]. Up to 2 mL of every sample was mixed with 4 mL (50 μg mL^-1^) of MeOH solution of DPPH. The mixture was vibrated and incubated for 30 min at 37°C in darkness, and the absorbance was measured at 517 nm using a spectrophotometer (Lambda 35; Perkin Elmer Co., Ltd., USA).

Scavenging activity of superoxide anion radical of fungal extract was determined by nitro-blue tetrazolium (NBT) reduction method [37]. In this process, xanthine oxidase produced O_2_^−^ and then reduced the yellow NBT^2+^ to produce blue formazan. The change speed of absorbance of the reactive solution was measured spectrophotometrically at 560 nm against a blank sample. The assay was conducted using a superoxide anion radical-scavenging kit (Nanjing Jiancheng Bioengineering Institute, China) according to the manufacturer’s protocol.

Hydroxyl radical-scavenging assay referenced the method of Škerget et al [38]. The reaction tube contained 0.6 mL of 20 mmol L^-1^ sodium salicylate, 2.0 mL of 1.5 mmol L^-1^ FeSO_4_, and 1 mL of sample solution, which were mixed and added to 1.4 mL of 6 mmol L^-1^ H_2_O_2_. The mixture was completely blended and reacted at 37°C for 1 h and then recorded its absorbance value at 510 nm.

The nitrite-scavenging activity was determined according to a method using Griess reagent [39]. Up to 2 mL of the sample was added to 3 mL of NaNO_2_ (5 μg mL^-1^), mixed, and adjusted pH to 1.2 with 0.1 mol L^-1^ HCl. The reaction mixture was incubated at 37°C for 1 h. Up to 5 mL of 2% acetic acid and 400 μL of Griess reagent (1:1 ratio of 1% sulfanilic acid in 30% acetic acid and 1% naphthylamine in 30% acetic acid) were added to the reaction solution. The mixture was shaken vigorously and incubated at room temperature for 15 min. The absorbance was recorded at 520 nm. Butylated hydroxytoluene (BHT) and vitamin C (Vc) were used as positive controls in the above tests.

Ferrous ion-chelating assay was evaluated by the modified method of Dinis et al. [40]. In brief, 1 mL of the sample was added in the tube, followed by 3.7 mL of 55% ethanol, 0.1 mL of 2 mmol L^-1^ FeCl_2_ and 0.2 mL 5 mmol L^-1^ ferrozine. The mixture was mixed and incubated at room temperature for 20 min. The absorbance was recorded at 562 nm against a blank sample. EDTA was used as positive control.

All the aforementioned measurements were calculated as follows:

$$\text{Scavenging rate}\left(\mathrm{SR}\right)\left(\%\right)=\left[\left(A_{0}-A_{i}\right)∕A_{0}\right]\times 100$$

where *A*_0_ is the absorbance without sample, and *A*_i_ is the absorbance in the presence of the samples or a positive substance.

### Total Phenolic and Total Flavonoid Content Assay

A modified method of Singleton et al. [41] was adopted for total phenolic content assay. Up to 1 mL of the sample (10 mg mL^-1^) was mixed with 1 mL of Folin–Ciocalteu’s phenol reagent, and the reaction solution was added with 1 mL of 35% Na_2_CO_3_ after 3 min and then with 10 mL of distilled water. The mixture was incubated at 180 rpm on a rotary shaker at 25°C for 1.5 h without light, followed by measurement of absorbance at 725 nm. Total phenolic contents were expressed as gallic acid equivalent (mg g^-1^) using the following equation based on the calibration curve: *y* = 6.5238*x* + 0.0084, *R* ^2^ = 0.9995. The standard curve was linear between 5 μg mL^-1^ and 100 μg mL^-1^ gallic acid. Besides, salidroside and *p*-tyrosol, two special compounds derived from *Rhodiola* plants, were screened to these endophytic fungal extract by RP-HPLC method according to the “Pharmacopeia of China” [7].

Total flavonoid content in fungal extract was estimated by colorimetry following the method of Ordoñez et al. [42]. In brief, 1 mL of extract (10 mg mL^-1^) was mixed with 4 mL of distilled water and 0.3 mL of 5% NaNO_3_. After 5 min, 0.3 mL of 10% AlCl_3_ was added and mixed well. After another 5 min, 2 mL of 1 mol L^-1^ NaOH was added. Finally, the volume reached up to 10 mL with distilled water and mixed well. Absorbance was measured at 510 nm. Total flavonoid contents were expressed as rutin (mg g^-1^) using the following equation based on the calibration curve: *y* = 8.23819*x* − 0.0095, *R* ^2^ = 0.9993. The standard curve was linear between 5 μg mL^-1^ and 100 μg mL^-1^ of rutin.

### Statistical Analysis

Diversity of fungal endophytes in three *Rhodiola* plants was evaluated by Shannon–Weiner Index (*H*′) and Evenness Index (*E*′) using the formulas: *H*′ = −∑(*Pi* × ln*Pi*) (*Pi* = *ni*/*N*, *ni* refers to the number of individual No. *i*, and *N* is the total number of individuals) and *E*′ = *H*′/ln *S* (*S* indicates the total number of species). The frequency of colonization (FC%) was calculated by the formula FC% = numbers of species/total number of species examined in every plant and all of the plants. Similarity index of fungal taxa between two of three *Rhodiola* species was determined by the formula *S* = 2*c*/(*a* + *b*) (*a* and *b* refer to the numbers of species in samples A and B, respectively, and *c* is their public number of species). All data in the antioxidant assay were expressed as the mean ± SD of triplicates (*n* = 3). Difference analysis with one-way ANOVA and post-hoc comparison with Least Significant Difference test were conducted with SPSS 18.0 for Windows (SPSS Inc., Chicago, USA). Drawing tools in Adobe Photoshop 8.0 and OriginPro 8.0 were used, and *p* < 0.05 was considered statistically significant.

## Results

### Isolates, Sequence Data, and Diversity

A total of 347 (isolation rate = 57.83%) endophytic fungi were isolated from 600 rhizome segments from three *Rhodiola* plants, including 126 (36.31%), 109 (31.41%), and 112 (32.28%) isolates from *Rc*, *Ra*, and *Rs*, respectively. They were designated into 180 representative morphotypes (71, 57, and 52 isolates from *Rc*, *Ra*, and *Rs*, respectively) based on cultural characteristics. These isolates were identified by rRNA gene ITS sequences or their potential related taxa. Except for 12 unidentified fungi without high similarity in the GenBank database, all other 168 isolates were categorized into level of species, genus, or family (S1 Table).

The results of diversity and similarity of 180 isolates from the three host plants showed at least 57 genera (28, 23, and 29 genera of endophytes from *Rc*, *Ra*, and *Rs*, respectively) (Table 1). In 57 genus taxa, 50 genera were affiliated to phyla Ascomycota, including 160 isolates (88.89%). Three genera contained five isolates (2.78%) belonging to Basidiomycota. Two genera included two isolates (1.11%) from Zygomycota. One genus contained one isolate (0.56%) affiliated to Glomeromycota. The exclusion of 12 unidentified fungi (6.67%) couldn't show that the endophytes were widely distributed. Shannon–Weiner diversity index (*H*′) was estimated based on taxonomic units or morphological characters. ITS sequences showed that *Rs* presented the highest fungal species diversity (3.086), followed by *Rc* (3.070) and *Ra* (2.843). However, *Rc* showed a higher Evenness index (*E*′) (0.921) than *Rs* (0.916) and *Ra* (0.907) (Table 1). Similarity index of the endophytic fungal taxa between *Ra* and *Rs* was 0.462, both of which were from the same regions. This result is higher than that between *Ra* and *Rc* (0.353) and that between *Rs* and *Rc* (0.246), which showed geographical preference of endophytic fungal distribution.

**Table 1:** Fungal endophytes from three *Rhodiola* species and their frequency of colonization (FC%).

Genus (stated in GenBank)	Phylum; Class; Order	Fungal isolate (Representative strains)	Isolate number				FC%			
			Rct	Rac	Rsc	Total	Rct	Rac	Rsc	total
*Ilyonectria*	Ascomycota; Sordariomycetes; Hypocreales	Rct18, Rct20, Rct21, Rct22, Rct29, Rct31, Rct32, and Rsc7	7	0	1	8	9.86	0	1.92	4.44
*Hypocrea*	Ascomycota; Sordariomycetes; Hypocreales	Rct8, Rct10, and Rac32	2	1	0	3	2.82	1.75	0	1.67
*Fusarium*	Ascomycota; Sordariomycetes; Hypocreales	Rct6, Rct7, Rct12, Rct13, Rct14, Rct26; Rac41, and Rsc8	6	1	1	8	8.45	1.75	1.92	4.44
*Neonectria*	Ascomycota; Sordariomycetes; Hypocreales	Rct23, Rct25, Rct27, Rct30, Rct71, and Rac36	5	1	0	6	7.04	1.75	0	3.33
*Thelonectria*	Ascomycota; Sordariomycetes; Hypocreales	Rct24	1	0	0	1	1.41	0	0	0.56
*Beauveria*	Ascomycota; Sordariomycetes; Hypocreales	Rct28	1	0	0	1	1.41	0	0	0.56
*Bionectria*	Ascomycota; Sordariomycetes; Hypocreales	Rct38	1	0	0	1	1.41	0	0	0.56
*Cordyceps*	Ascomycota; Sordariomycetes; Hypocreales	Rct61	1	0	0	1	1.41	0	0	0.56
*Trichoderma*	Ascomycota; Sordariomycetes; Hypocreales	Rac43 and Rsc4	0	1	1	2	0	1.75	1.92	1.11
*Gibberella*	Ascomycota; Sordariomycetes; Hypocreales	Rac15, Rac27;Rsc58, and Rsc71	0	2	2	4	0	3.51	3.85	2.22
*Lecythophora*	Ascomycota; Sordariomycetes;Coniochaetales	Rct47	1	0	0	1	1.41	0	0	0.56
*Coniochaeta*	Ascomycota; Sordariomycetes;Coniochaetales	Rct69	1	0	0	1	1.41	0	0	0.56
Phomopsis	Ascomycota; Sordariomycetes; Diaporthales	Rac6, Rac9, Rac44, Rac59, Rac60, and Rac61	0	6	0	6	0	10.53	0	3.33
*Cytospora*	Ascomycota; Sordariomycetes; Diaporthales	Rsc29	0	0	1	1	0	0	1.92	0.56
*Biscogniauxia*	Ascomycota; Sordariomycetes; Xylariales	Rsc5	0	0	1	1	0	0	1.92	0.56
*Pestalotiopsis*	Ascomycota; Sordariomycetes; Xylariales	Rsc44	0	0	1	1	0	0	1.92	0.56
*Chaetomium*	Ascomycota; Sordariomycetes; Sordariales	Rac51 and Rsc3	0	1	1	2	0	1.75	1.92	1.11
Sordariomycetes	Ascomycota; Sordariomycetes	Rac37	0	1	0	1	0	1.75	0	0.56
*Saccharicola*	Ascomycota; Dothideomycetes; Pleosporales	Rct1, Rct2, and Rct4	3	0	0	3	4.23	0	0	1.67
*Ulocladium*	Ascomycota; Dothideomycetes; Pleosporales	Rct15 and Rct36	2	0	0	2	2.82	0	0	1.11
*Stagonosporopsis*	Ascomycota; Dothideomycetes; Pleosporales	Rct33	1	0	0	1	1.41	0	0	0.56
*Alternaria*	Ascomycota; Dothideomycetes; Pleosporales	Rct35, Rct48, Rct50, Rct52, Rct54, Rct55, Rct56, Rac30, Rac58, Rsc40, Rsc41, and Rsc59	7	2	3	12	9.86	3.51	5.77	6.67
*Phoma*	Ascomycota; Dothideomycetes; Pleosporales	Rct42, Rac11, Rac54, Rac55, and Rsc43	1	3	1	5	1.41	5.26	1.92	2.78
*Peyronellaea*	Ascomycota; Dothideomycetes; Pleosporales	Rct53	1	0	0	1	1.41	0	0	0.56
*Epicoccum*	Ascomycota; Dothideomycetes; Pleosporales	Rac25, Rac29, Rac71, and Rsc2	0	3	1	4	0	5.27	1.92	2.22
*Phaeosphaeria*	Ascomycota; Dothideomycetes; Pleosporales	Rac47 and Rsc62	0	1	1	2	0	1.75	1.92	1.11
*Leptosphaeria*	Ascomycota; Dothideomycetes; Pleosporales	Rsc20, Rsc21, Rsc27, Rsc47, Rsc49, Rsc64, Rsc65, Rsc72 and Rsc73	0	0	9	9	0	0	17.31	5.00
*Cladosporium*	Ascomycota; Dothideomycetes; Capnodiales	Rct17 and Rac52	1	1	0	2	1.41	1.75	0	1.11
*Trimmatostroma*	Ascomycota; Dothideomycetes; Capnodiales	Rsc18 and Rsc55	0	0	2	2	0	0	3.85	1.11
*Dothichiza*	Ascomycota; Dothideomycetes; Dothideales	Rct49	1	0	0	1	1.41	0	0	0.56
Dothideomycetes	Ascomycota; Dothideomycetes	Rsc24, Rsc53, and Rsc57	0	0	3	3	0	0	5.77	1.67
*Botrytis*	Ascomycota; Leotiomycetes; Helotiales	Rct3, Rct41, Rct57, Rct62, and Rct68	5	0	0	5	7.04	0	0	2.78
*Botryotinia*	Ascomycota; Leotiomycetes; Helotiales	Rct11, Rct58, Rct59, and Rct70	4	0	0	4	5.63	0	0	2.22
*Cadophora*	Ascomycota; Leotiomycetes; Helotiales	Rct16, Rct34, Rct39, Rct46, and Rsc38	4	0	1	5	5.63	0	1.92	2.78
*Leptodontium*	Ascomycota; Leotiomycetes; Helotiales	Rsc66, Rsc67, Rsc68, and Rsc70	0	0	4	4	0	0	7.69	2.22
*Tetracladium*	Ascomycota; Leotiomycetes; Helotiales	Rct51 and Rct67	2	0	0	2	2.82	0	0	1.11
*Phialocephala*	Ascomycota; Leotiomycetes; Helotiales	Rac2, Rac3, Rac26, Rac56, Rac57, Rac63, Rac66, and Rsc22	0	7	1	8	0	12.28	1.92	4.44
*Cryptosporiopsis*	Ascomycota; Leotiomycetes; Helotiales	Rac4, Rac14, Rac38, Rac40, Rac73, and Rac74	0	6	0	6	0	10.53	0	3.33
*Lachnum*	Ascomycota; Leotiomycetes; Helotiales	Rac12, Rac76, and Rsc13	0	2	1	3	0	3.51	1.92	1.67
*Pseudaegerita*	Ascomycota; Leotiomycetes; Helotiales	Rsc23	0	0	1	1	0	0	1.92	0.56
*Varicosporium*	Ascomycota; Leotiomycetes; Helotiales	Rsc33	0	0	1	1	0	0	1.92	0.56
*Proliferodiscus*	Ascomycota; Leotiomycetes; Helotiales	Rsc39	0	0	1	1	0	0	1.92	0.56
Rhytismataceae	Ascomycota; Leotiomycetes; Rhytismatales	Rct43	1	0	0	1	1.41	0	0	0.56
Leotiomycetes	Ascomycota; Leotiomycetes	Rsc36, Rsc46, and Rsc52	0	0	3	3	0	0	5.77	1.67
*Phialophora*	Ascomycota; Eurotiomycetes; Chaetothyriales	Rct40, Rct44, and Rct45	3	0	0	3	4.23	0	0	1.67
*Exophiala*	Ascomycota; Eurotiomycetes; Chaetothyriales	Rsc15	0	0	1	1	0	0	1.92	0.56
*Capronia*	Ascomycota; Eurotiomycetes; Chaetothyriales	Rsc42	0	0	1	1	0	0	1.92	0.56
*Penicillium*	Ascomycota; Eurotiomycetes; Eurotiales	Rct5, Rct37, Rct63, Rac39, Rsc11, and Rsc31	3	1	2	6	4.23	1.75	3.85	3.33
*Aspergillus*	Ascomycota; Eurotiomycetes; Eurotiales	Rct9, Rct64, Rct65, Rct66; Rac8, Rac62, and Rac77, Rac79	4	4	0	8	5.63	7.02	0	4.44
*Microsphaeropsis*	Ascomycota; Loculoascomycetes; Dothideales	Rac53	0	1	0	1	0	1.75	0	0.56
*Ceratobasidium*	Basidiomycota; Agaricomycetes; Cantharellales	Rac69, Rac81, and Rac85	0	3	0	3	0	5.27	0	1.67
*Rhizoctonia*	Basidiomycota; Agaricomycetes; Cantharellales	Rsc51	0	0	1	1	0	0	1.92	0.56
*Coprinellus*	Basidiomycota; Agaricomycetes; Agaricales	Rsc45	0	0	1	1	0	0	1.92	0.56
*Umbelopsis*	Zygomycota; Mortierellaceae; Mucorales	Rac18	0	1	0	1	0	1.75	0	0.56
*Mucor*	Zygomycota; Zygomycetes; Mucorales	Rct60	1	0	0	1	1.41	0	0	0.56
*Entrophospora*	Glomeromycota; Glomeromycetes; Diversisporales	Rac88	0	1	0	1	0	1.75	0	0.56
Unidentified fungi		Rct19; Rac23, Rac31, Rac45, Rac46, Rac50, Rac65, Rac67; Rsc1, Rsc9, Rsc30, and Rsc69	1	7	4	12	1.41	12.28	7.69	6.67
Individual number			71	57	52	180				
Shannon index (*H*′)			3.070	2.843	3.086					
Evenness index (*E*′)			0.921	0.907	0.916					

*Rc*, *Rhodiola crenulata; Ra*, *R*. *angusta* and *Rs*, *R*. *sachalinensis*; Rct: the isolates from *Rc* in Tibet; Rac and Rsc: the isolates from *Ra* and *Rs* in Changbai Mountain.

Further taxonomic analysis showed that only isolate Rac88 from host *Ra* was affiliated to phylum Glomeromycota and placed in the genus *Entrophospora*. Isolate Rct60 was closely matched to the sequence from *Mucor hiemalis* (99%), and isolate Rac18 exhibited only 78% identity with *Umbelopsis* sp., which was only allocated to the order Mucorales in Zygomycota. Within Basidiomycota, three isolates, namely, Rac69, Rac81, and Rac85, all from the same host *Ra*, were closely related to the sequences from *Ceratobasidium* sp. Isolates Rsc45 and Rsc51, both from *Rs*, were affiliated to *Coprinellus xanthothrix* (99%) and *Rhizoctonia solani* (100%), respectively (S1 Table).

Most endophytic fungi from the three *Rhodiola* plants belong to the three classes in phylum Ascomycota, namely, Sordariomycetes, Dothideomycetes, and Leotiomycetes, and their FC% was 27.22% (49/180), 26.11% (47/180), and 24.44% (44/180), respectively (Table 1). In Sordariomycetes, 35 isolates were assigned to 10 genera in Hypocreales, and only one or two isolates (Rct47, Rct69, Rsc29, Rsc5, Rsc44, Rac51, or Rsc3) were placed in six genera (*Lecythophora*, *Coniochaeta*, *Cytospora*, *Biscogniauxia*, *Pestalotiopsis*, and *Chaetomium*) belonging to Coniochaetales, Diaporthales, Xylariales, and Sordariales (Table 1), respectively. Six isolates (Rac6, Rac9, Rac44, Rac59, Rac60, and Rac61) from the same host *Ra* belonged to Diaporthales and were close to *Phomopsis* spp. The isolate Rac37 was placed in Sordariomycetes without any similar defined sequence under the level of class (S1 Table). Within Dothideomycetes, 82.98% (39) of the isolates belonged to Pleosporales, and 12 isolates were closely matched to the sequences from *Alternaria* spp. Nine isolates were closely related to the members of *Leptosphaeria*, which was the dominant fungus of the host *Rs* (Table 1). In the order Capnodiales, four isolates (Rct17, Rac52, Rsc18, and Rsc55) were assigned to genera *Cladosporium* and *Trimmatostroma*. Only isolate Rct49 was affiliated to genus *Dothichiza* in the order Dothideales. The other isolates (Rsc24, Rsc53, and Rsc57) were placed in Dothideomycetes without any similar defined sequence under the level of class. Within Leotiomycetes, the order Helotiales included 40 isolates distributed in at least 11 genera (Table 1). Isolate Rct43 exhibited only 92% identity with the family Rhytismataceae, and isolates Rsc36, Rsc46, and Rsc52 were only placed in Leotiomycetes according to the aligned results in BLAST search. Among the 19 isolates in class Eurotiomycetes, two common genera *Penicillium* and *Aspergillus* (Eurotiales) covered 14 isolates. Five isolates belonging to Chaetothyriales were close to *Phialophora* (Rct40, Rct44, and Rct45), *Exophiala* (Rsc15), and *Capronia* (Rsc42) with high identity (S1 Table). Another isolate from host *Ra*, Rac53, exhibited a well-supported sequence alignment with *Microsphaeropsis arundinis* (99%) in phylum Ascomycota.

### Assay of Antioxidant Activity of Culturable Strains

This study also aimed to obtain isolates with high antioxidant activities from *Rhodiola* plants. The results show that the mycelia extracts had higher antioxidant activity than those of filtrates. The primary screening results of DPPH radical-scavenging assay also showed that SRs of 114 isolates (63.33%) were >50% in 180 representative fungi. SRs between 50% and 60%, 60% to 70%, 70% to 80%, 80% to 90%, and 90% to 100% were found in 47 (26.11%), 29 (16.11%), 19 (10.56%), 14 (7.78%), and 5 (2.78%) isolates, respectively (Table 2). The share of isolates with high antioxidant activity (SR > 50%) was similar between *Ra* (73.6%) and *Rs* (73.1%), which exhibited more than *Rc* (47.9%) (Table 2).

**Table 2:** Number of endophytic fungi with different DPPH radical-scavenging rates.

^a)^ SR	Number of endophytic fungi			
	^b)^ *Rc*	^c)^ *Ra*	^d)^ *Rs*	Total (%)
<50%	37	15	14	66 (36.67)
≥50%	34	42	38	114 (63.33)
50%–60%	14	18	15	47 (26.11)
60%–70%	5	12	12	29 (16.11)
70%–80%	6	6	7	19 (10.56)
80%–90%	6	5	3	14 (7.78)
90%–100%	3	1	1	5 (2.78)

^a)^ SR, DPPH radicals-scavenging rates

^b)^ *Rc*, *Rhodiola crenulata*

^c)^ *Ra*, *R*. *angusta*

^d)^ *Rs*, *R*. *sachalinensis*.

Five isolates (SR > 90%) (Rct45, Rct63, Rct64, Rac76, and Rsc57) were further selected for antioxidant activity assay at different concentrations (1 mg mL^-1^ to 10 mg mL^-1^) using five methods. Their EC_50_ values were obtained by interpolation from linear regression analysis (Table 3), which showed that each isolate displayed one or several high activities in five antioxidant assays. Except for Rct45, all other fungal EC_50_ values of DPPH radical-scavenging assay were slightly higher than that of Vc (1.28 mg mL^-1^) and BHT (3.13 mg mL^-1^), which exhibited lower DPPH radical-scavenging activities. However, the EC_50_ value of Rct45 was 1.54 mg mL^-1^, which was higher than that of Vc but lower than that of BHT. In summary, EC_50_ values indicated that all five isolates were potential fungi with high DPPH radical-scavenging activities. The highest hydroxyl radical-scavenging activity (EC_50_ = 1.41 mg mL^-1^), followed by Rct64 (3.17 mg mL^-1^), Rac76 (13.99 mg mL^-1^), Rct63 (35.75 mg mL^-1^), and Rsc57 (36.59 mg mL^-1^), and the EC_50_ values of positive control Vc and BHT were 13.35 and 7.09 mg mL^-1^. Thus, Rct45 and Rct64 were potential fungi on hydroxyl radical-scavenging activity. The EC_50_ values of scavenging activities on superoxide radical were 0.24, 0.92, 3.03, 5.36, and 6.70 mg mL^-1^ for Rct45, Rac76, Rct64, Rct63, and Rsc57, respectively, which showed that Rct45 and Rac76 exhibited good superoxide radical activity compared with Vc (0.47 mg mL^-1^) and BHT (0.98 mg mL^-1^) (Table 3). In nitrite-scavenging assay, the EC_50_ values of Rac76 and Rsc57 were lower than those of Rct45 (14.85 mg mL^-1^), Rct63 (12.14 mg mL^-1^), and Rct64 (14.46 mg mL^-1^) but higher than those of Vc (6.82 mg mL^-1^) and BHT (8.42 mg mL^-1^). The EC_50_ value of Rct64 was 3.03 mg mL^-1^ according to the Ferrous ion-chelating assay, followed by EDTA-2Na (9.68 mg mL^-1^), Rsc57 (12.06 mg mL^-1^), Rct63 (18.22 mg mL^-1^), Rac76 (28.89 mg mL^-1^), and Rct45 (35.95 mg mL^-1^), indicating that Rct64 exhibited a rather prominent ferrous ion-chelating activity.

**Table 3:** EC50 values from five antioxidant activity assays and contents of total phenolics and total flavonoids from five isolates of *Rhodiola* rhizomes.

	EC_50_ value (mg extract per mL)					Total phenols (mg g^-1^)	Total flavonoids (mg g^-1^)
	DPPH radicals	Hydroxyl radicals	Superoxide radicals	Nitrite radicals	Ferrous ions		
Rct45	1.54 ± 0.007^f^	1.41 ± 0.007^g^	0.24 ± 0.007^g^	14.85 ± 0.022^a^	35.95 ± 0.050^a^	24.64 ± 0.001^b^	23.90 ± 0.001^a^
Rct63	3.54±0.013^d^	35.75±0.016^b^	5.36±0.011^b^	12.14±0.016^c^	18.22±0.069^c^	12.31±0.001^d^	23.67±0.002^b^
Rct64	4.66±0.038^c^	3.17±0.020^f^	3.03±0.007^c^	14.46±0.013^b^	3.03±0.067^f^	24.75±0.002^a^	12.15±0.000^d^
Rac76	7.33±0.011^a^	13.99±0.173^c^	0.92±0.007^e^	9.14±0.011^e^	28.89±0.060^b^	11.96±0.001^e^	23.39±0.001^c^
Rsc57	6.58±0.029^b^	36.59±0.091^a^	6.70±0.027^a^	9.82±0.033^d^	12.06±0.020^d^	23.09±0.002^c^	11.92±0.001^e^
Vc	1.28±0.018^g^	13.35±0.044^d^	0.47±0.011^f^	6.82±0.047^g^	—	—	—
BHT	3.13 ± 0.009^e^	7.09 ± 0.033^e^	0.98 ± 0.011^d^	8.42 ± 0.107^f^	—	—	—
EDTA-2Na	—	—	—	—	9.68 ± 0.144^e^	—	—

Rct: the isolates from *Rc* in Tibet; Rac and Rsc: the isolates from *Ra* and *Rs* in Changbai Mountain; Vc: Vitamin C; BHT: Butylated hydroxytoluene; EC_50_ value, effective concentration at which antioxidant activity was 50% obtained by interpolation from linear regression analysis; absorbance was 0.5 for 1,1-diphenyl-2-picrylhydrazyl (DPPH), hydroxyl radicals (OH), superoxide radicals (O_2_^−^), and nitrite (NO_2_^−^) scavenged by 50%. Ferrous ions were chelated by 50%. Each value was mean ± SD (*n* = 3). Means with different letters within a column were significantly different (*p* < 0.05).

Total phenols and total flavonoids, which are main components closely related to antioxidant activity, were determined. The results indicated statistical differences among different fungal extracts. The highest content of total phenols was found in extracts from Rct64 (24.75 mg g^-1^), followed by Rct45 (24.64 mg g^-1^), Rsc57 (23.09 mg g^-1^), Rct63 (12.31 mg g^-1^), and Rac76 (11.96 mg g^-1^). The total flavonoids were in the order of Rct45 > Rct63 > Rac76 > Rct64 > Rsc57 (Table 3). Thus, Rct45 and Rct64 exhibited the highest phenols and flavonoids, which were responsible for higher effectiveness in scavenging radicals and chelating ferrous ions.

**Fig 2:**
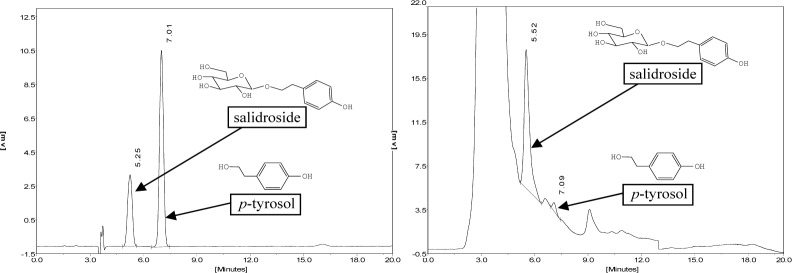
HPLC chromatogram of standard salidroside and *p*-tyrosol and Rac12 (*Lachnum* sp.) extract.

In the current study, the chemicals of mycelia extracts from endophytic fungi were also determined preliminarily by RP-HPLC method, which showed that the strain Rac12 (*Lachnum* sp.) with SR = 82.20% by DPPH radical-scavenging assay produced salidroside (0.131 ± 0.009 mg g^-1^) and *p*-tyrosol (0.113 ± 0.010 mg g^-1^) (Fig. 2) which were the main active compounds of *Rhodiola* plants. The results suggested that the endophytic fungi from *Rhodiola* spp. are potential sources of natural antioxidant products.

## Discussion

Most endophytic fungi have been studied from terrestrial plants distributed at low altitudes and temperate regions [43], which may be ascribed to the convenience of sample collection, biodiversity, extensive use, and clear background of plants, thereby disregarding the research of alpine plants [20]. However, those plants in relatively extreme environments are promising sources of fungal diversity and novel products [44]. Studies on these plants, particularly endophytic fungi related to antioxidants, have rarely been conducted [27]. In the present study, endophytic fungi and their antioxidant activities were investigated for the first time from *Rhodiola* plants, which are known for high antioxidant activities. The results showed that endophytic fungi are very abundant and diverse in the rhizomes of *Rhodiola* spp., and 180 representative isolates are distributed in at least in 57 genera affiliated to four fungal phyla. A large percentage of fungi exhibiting strong antioxidant activity indicated that these plants and their endophytes could be potential sources of novel natural antioxidants.

The specificity and selectivity between endophytic fungi and host plants remain unclear because of fungal diversity and limitation of current research methods [45]. However, many investigations have demonstrated this universal phenomenon [26,46]. In the present study, the specificity and preference were displayed in three aspects: (1) Endophytic fungi demonstrated species-specific isolates with their hosts, including 17, 7, and 15 isolates exclusively detected specific in *Rc*, *Ra*, and *Rs* plants, respectively (Table 1). Some of these isolates have been reported as endophytes [17,23,43], but all were reported from *Rhodiola* plants for the first time. Other fungi, obtained from all three plants belonging to four genera, including *Fusarium* (4.44%), *Alternaria* (6.67%), *Phoma* (2.78), and *Penicillium* (3.33%), have been reported as endophytes and pathogens in many plants [47,48]. (2) These fungi exhibited regional specificity with their hosts. The *Rc* plants were collected from Tibetan Plateau in southwestern China, whereas *Ra* and *Rs* plants were obtained from Changbai Mountain in northeast China. These two regions were >4,000 km apart (Fig. 1). Their different environmental and biological factors likely influenced the fungal species compositions in hosts [49], which lead to special endophytic composition in the host of the given area. For example, seven fungal genera (*Trichoderma*, *Gibberella*, *Chaetomium*, *Epicoccum*, *Phaeosphaeria*, *Phialocephala*, and *Lachnum*) were detected from both *Ra* and *Rs* plants but not isolated from *Rc* which obtained exclusively 17 genera isolates. However, only four (*Hypocrea*, *Neonectria*, *Cladosporium*, and *Aspergillus*) were found between *Rc* and *Ra*, and only two generic fungus (*Ilyonectria*) between *Rc* and *Rs* (Table 1). Notably, some endophytic fungi demonstrated distinctive regional features. For instance, Rct28 and Rct61 from *Rc* were highly similar to *Beauveria bassiana* (100%) and *Ophiocordyceps crassispora* (100%) (S1 Table), and the former was the anamorph of *Ophiocordyceps bassiana* [50]. *Ophiocordyceps* spp. are described as endophytes and entomogenous fungi from Asia, particularly from Nepal and southwest China [50,51]. These species are dominant fungal members in caterpillar fungus, a well-known traditional Chinese medicine mainly produced in the Qinghai–Tibet plateau in southwestern China [52]. (3) Functional tendency of antioxidant activity exists between endophytic fungi and their hosts. Strobel [27] suggested that plants with medicinal values provide the most feasible opportunities to obtain novel endophytic fungi for new medicinal products. In the current study, most of the endophytic fungi indicated antioxidant activity to some extent (Table 2). Five isolates (Rct45, Rct63, Rct64, Rac76, and Rsc57) with strong antioxidant activities were isolated from *Rhodiola* plants with high antioxidant capacities. Similar studies support the antioxidant preference between endophytes and their hosts [29,53,54]. For example, several endophytic fungi with strong antioxidant activities were isolated from medicinal plants (such as *Melodinus suaveolens* and *Nerium oleander*) with high antioxidant capacities according to the research of Yang et al. [54]. However, the hypothesis of specificity and selectivity needs further investigation because our samples were inadequate, the endophyte community was dynamic, the culture-independent fungi were not considered, and many factors (seasonal changes, host age, etc.) affected the species composition [55].

Studies showed that phenolic and flavonoid compounds were dominant antioxidant components in natural plants [56]. *Rhodiola* plants contained salidrosides, *p*-tyrosol, and rosavins [14]. However, no comparative investigation has been performed for their endophytes. Surprisingly, the fermentation broth of Rac12 was confirmed to produce salidroside and *p*-tyrosol by HPLC assay in this study. These results suggest that versatile fungal endophytes may produce many novel antioxidant products, including the same bioactive chemicals as those of their hosts. The important relationship between Rac12 and its host can be elucidated by illustrating the biosynthetic pathway in two organisms, and the production from Rac12 will be significantly increased by screening industrial mutants and optimizing the fermentation process in future studies.

Accordingly, endophytic fungi were highly abundant in *Rhodiola* plants. However, their mutual relationship, ecological function, and relevance of metabolic pathways need extensive investigation. These species are potential viable sources for exploring novel natural antioxidant products.

## Supporting Information

S1 Table Taxon designation and GenBank accession numbers of fungal endophytes from *Rhodiola* rhizomes. (DOC)
(DOCX)
