# Supplementary material for: Diversity and Antioxidant Activity of Culturable Endophytic Fungi from Alpine Plants of Rhodiola crenulata, R. angusta, and R. sachalinensis
Source: PLoS One. 2015 Mar 13;10(3):e0118204. doi: 10.1371/journal.pone.0118204 (PMC4359136; doi:10.1371/journal.pone.0118204)
Supplement: S1 Table — (DOC) (DOCX) [file pone.0118204.s001.docx]

**Table S1.** Taxon designation and GenBank accession numbers of fungal endophytes from *Rhodiola* rhizomes.

| **Code** | **Reference species (RS)** | **Closest species match (Accession No.)** | **% Identity** | **Accession No. of RS** | **Host** |
| --- | --- | --- | --- | --- | --- |
| 1 | Rct1 | *Saccharicola bicolor* ( AF455415 ) | 99 | KJ542191 | Rc |
| 2 | Rct2 | *Saccharicola bicolor* ( AF455415 ) | 99 | KJ542192 | Rc |
| 3 | Rct3 | *Botrytis byssoidea* ( FJ914712 ) | 99 | KJ542193 | Rc |
| 4 | Rct4 | *Saccharicola bicolor* ( AF455415 ) | 99 | KJ542194 | Rc |
| 5 | Rct5 | *Penicillium chrysogenum* ( JN585943 ) | 100 | KJ542195 | Rc |
| 6 | Rct6 | [*Fusarium oxysporum*](http://blast.ncbi.nlm.nih.gov/Blast.cgi#alnHdr_411146009) ( KF494093 ) | 100 | KJ542196 | Rc |
| 7 | Rct7 | *Fusarium* [*oxysporum*](http://blast.ncbi.nlm.nih.gov/Blast.cgi#alnHdr_525469675) ( KF313101 ) | 100 | KJ542197 | Rc |
| 8 | Rct8 | [*Hypocrea pachybasioides*](http://blast.ncbi.nlm.nih.gov/Blast.cgi#alnHdr_33090021) ( AY240844 ) | 100 | KJ542198 | Rc |
| 9 | Rct9 | *Aspergillus* *niger* ( KF496081 ) | 100 | KJ542199 | Rc |
| 10 | Rct10 | [*Hypocrea lixii*](http://blast.ncbi.nlm.nih.gov/Blast.cgi#alnHdr_396576522) ( JX244280 ) | 100 | KJ542200 | Rc |
| 11 | Rct11 | [*Botryotinia fuckeliana*](http://blast.ncbi.nlm.nih.gov/Blast.cgi#alnHdr_304263464) ( HM989942 ) | 99 | KJ542201 | Rc |
| 12 | Rct12 | *Fusarium* *lacertarum* ( JF740923 ) | 100 | KJ542202 | Rc |
| 13 | Rct13 | [*Fusarium acuminatum*](http://blast.ncbi.nlm.nih.gov/Blast.cgi#alnHdr_556904148) ( KC916647 ) | 100 | KJ542203 | Rc |
| 14 | Rct14 | [*Fusarium redolens*](http://blast.ncbi.nlm.nih.gov/Blast.cgi#alnHdr_529280073) ( KC924920 ) | 100 | KJ542204 | Rc |
| 15 | Rct15 | [*Ulocladium* *multiforme*](http://blast.ncbi.nlm.nih.gov/Blast.cgi#alnHdr_148357893) ( EU330458 ) | 100 | KJ542205 | Rc |
| 16 | Rct16 | *Cadophora* *melinii* (JN689950) | 99 | KJ542206 | Rc |
| 17 | Rct17 | *Cladosporium cladosporioides* ( JX535135 ) | 100 | KJ542207 | Rc |
| 18 | Rct18 | *IIynoectria* *macrodidym*a ( JX231163 ) | 100 | KJ542208 | Rc |
| 19 | Rct20 | [*Ilyonectria macrodidyma*](http://blast.ncbi.nlm.nih.gov/Blast.cgi#alnHdr_387773632) ( JQ846082 ) | 100 | KJ542209 | Rc |
| 20 | Rct21 | Ilyonectria [*macrodidyma*](http://blast.ncbi.nlm.nih.gov/Blast.cgi#alnHdr_486170915) ( JX231163 ) | 100 | KJ542210 | Rc |
| 21 | Rct22 | [*Ilyonectria macrodidyma*](http://blast.ncbi.nlm.nih.gov/Blast.cgi#alnHdr_486170915) ( JX231163 ) | 100 | KJ542211 | Rc |
| 22 | Rct23 | [*Neonectria ramulariae*](http://blast.ncbi.nlm.nih.gov/Blast.cgi#alnHdr_345447657) ( JF735314 ) | 99 | KJ542212 | Rc |
| 23 | Rct24 | [*Thelonectria veuillotiana*](http://blast.ncbi.nlm.nih.gov/Blast.cgi#alnHdr_390118211) ( JQ734922 ) | 99 | KJ542213 | Rc |
| 24 | Rct25 | [*Neonectria ramulariae*](http://blast.ncbi.nlm.nih.gov/Blast.cgi#alnHdr_345447657) ( JF735314 ) | 99 | KJ542214 | Rc |
| 25 | Rct26 | *Fusarium* *tricinctum* ( JQ676180 ) | 100 | KJ542215 | Rc |
| 26 | Rct27 | [*Neonectria ramulariae*](http://blast.ncbi.nlm.nih.gov/Blast.cgi#alnHdr_345447657) ( JF735314 ) | 99 | KJ542216 | Rc |
| 27 | Rct28 | *Beauveria bassiana* ( KC753398 ) | 100 | KJ542217 | Rc |
| 28 | Rct29 | [*Ilyonectria macrodidyma*](http://blast.ncbi.nlm.nih.gov/Blast.cgi#alnHdr_387773632) ( JQ846082 ) | 100 | KJ542218 | Rc |
| 29 | Rct30 | [*Neonectria ramulariae*](http://blast.ncbi.nlm.nih.gov/Blast.cgi#alnHdr_345447657) ( JF735314 ) | 99 | KJ542219 | Rc |
| 30 | Rct31 | [*Ilyonectria* sp.](http://blast.ncbi.nlm.nih.gov/Blast.cgi#alnHdr_345447661)( JF735318 ) | 98 | KJ542220 | Rc |
| 31 | Rct32 | [*Ilyonectria robusta*](http://blast.ncbi.nlm.nih.gov/Blast.cgi#alnHdr_345447608) ( JF735265 ) | 99 | KJ542221 | Rc |
| 32 | Rct33 | [*Stagonosporopsis cucurbitacearum*](http://blast.ncbi.nlm.nih.gov/Blast.cgi#alnHdr_343114005) ( JN253542) | 99 | KJ542222 | Rc |
| 33 | Rct34 | [*Cadophora melinii*](http://blast.ncbi.nlm.nih.gov/Blast.cgi#alnHdr_355343839) ( JN689950 ) | 99 | KJ542223 | Rc |
| 34 | Rct35 | *Alternaria* *alternata* ( KF438091 ) | 100 | KJ542224 | Rc |
| 35 | Rct36 | [*Ulocladium consortiale*](http://blast.ncbi.nlm.nih.gov/Blast.cgi#alnHdr_498541790) ( KC577270 ) | 100 | KJ542225 | Rc |
| 36 | Rct37 | [*Penicillium* chrysogenum](http://blast.ncbi.nlm.nih.gov/Blast.cgi#alnHdr_315201004)( JN585943 ) | 100 | KJ542226 | Rc |
| 37 | Rct38 | [*Bionectria ochroleuca*](http://blast.ncbi.nlm.nih.gov/Blast.cgi#alnHdr_315200933) ( HQ637283 ) | 100 | KJ542227 | Rc |
| 38 | Rct39 | [*Cadophora*](http://blast.ncbi.nlm.nih.gov/Blast.cgi#alnHdr_315200952) *melinii* ( JN689950 ) | 99 | KJ542228 | Rc |
| 39 | Rct40 | [*Phialophora* sp.](http://blast.ncbi.nlm.nih.gov/Blast.cgi#alnHdr_307548380) ( AB190386 ) | 98 | KJ542229 | Rc |
| 40 | Rct41 | *Botrytis byssoidea* ( FJ914712 ) | 100 | KJ542230 | Rc |
| 41 | Rct42 | [*Phoma* sp*.*](http://blast.ncbi.nlm.nih.gov/Blast.cgi#alnHdr_197085531) ( AB333774 ) | 97 | KJ542231 | Rc |
| 42 | Rct43 | [Rhytismataceae sp.](http://blast.ncbi.nlm.nih.gov/Blast.cgi#alnHdr_326319934) ( FR837916 ) | 92 | KJ542232 | Rc |
| 43 | Rct44 | [*Phialophora*](http://blast.ncbi.nlm.nih.gov/Blast.cgi#alnHdr_307548380) sp. ( AB190386 ) | 98 | KJ542233 | Rc |
| 44 | Rct45 | [*Phialophora* sp.](http://blast.ncbi.nlm.nih.gov/Blast.cgi#alnHdr_307548380) ( AB190386 ) | 98 | KJ542234 | Rc |

**Table S1.** Continued.

| **Code** | **Reference species (RS)** | **Closest species match (Accession No.)** | **% Identity** | **Accession No. of RS** | **Host** |
| --- | --- | --- | --- | --- | --- |
| 45 | Rct46 | *Cadophora* sp. ( KC514850 ) | 97 | KJ542235 | Rc |
| 46 | Rct47 | [*Lecythophora* sp.](http://blast.ncbi.nlm.nih.gov/Blast.cgi#alnHdr_315200946) ( HQ637296 ) | 98 | KJ542236 | Rc |
| 47 | Rct48 | [*Alternaria*](http://blast.ncbi.nlm.nih.gov/Blast.cgi#alnHdr_558854124) *arborescens* ( KC707557 ) | 100 | KJ542237 | Rc |
| 48 | Rct49 | *Dothichiza* sp. ( JX188157 ) | 97 | KJ542238 | Rc |
| 49 | Rct50 | [*Alternaria*](http://blast.ncbi.nlm.nih.gov/Blast.cgi#alnHdr_558854124) *tenuissima* ( KF494005 ) | 100 | KJ542239 | Rc |
| 50 | Rct51 | *Tetracladium setigerum* ( HQ647302 ) | 97 | KJ542240 | Rc |
| 51 | Rct52 | [*Alternaria*](http://blast.ncbi.nlm.nih.gov/Blast.cgi#alnHdr_558854124) *alternata* ( KJ767515 ) | 100 | KJ542241 | Rc |
| 52 | Rct53 | [*Peyronellaea arachidicola*](http://blast.ncbi.nlm.nih.gov/Blast.cgi#alnHdr_413968331) ( JX898682 ) | 100 | KJ542242 | Rc |
| 53 | Rct54 | [*Alternaria*](http://blast.ncbi.nlm.nih.gov/Blast.cgi#alnHdr_558854124) *tenuissima* ( KF494005 ) | 100 | KJ542243 | Rc |
| 54 | Rct55 | [*Alternaria alternata*](http://blast.ncbi.nlm.nih.gov/Blast.cgi#alnHdr_553303883) ( KJ526175 ) | 100 | KJ542244 | Rc |
| 55 | Rct56 | [*Alternaria alternata*](http://blast.ncbi.nlm.nih.gov/Blast.cgi#alnHdr_553303883) ( KJ526175 ) | 100 | KJ542245 | Rc |
| 56 | Rct57 | [*Botrytis elliptica*](http://blast.ncbi.nlm.nih.gov/Blast.cgi#alnHdr_373809852) ( AB693905 ) | 99 | KJ542246 | Rc |
| 57 | Rct58 | [*Botryotinia fuckeliana*](http://blast.ncbi.nlm.nih.gov/Blast.cgi#alnHdr_540070776) ( KF532992 ) | 99 | KJ542247 | Rc |
| 58 | Rct59 | [*Botryotinia fuckeliana*](http://blast.ncbi.nlm.nih.gov/Blast.cgi#alnHdr_540070776) ( KF532992 ) | 99 | KJ542248 | Rc |
| 59 | Rct60 | [*Mucor hiemalis*](http://blast.ncbi.nlm.nih.gov/Blast.cgi#alnHdr_302121732) ( KJ589612 ) | 99 | KJ542249 | Rc |
| 60 | Rct61 | [*Ophiocordyceps crassispora*](http://blast.ncbi.nlm.nih.gov/Blast.cgi#alnHdr_29466986) ( AB067714 ) | 100 | KJ542250 | Rc |
| 61 | Rct62 | [*Botrytis byssoidea*](http://blast.ncbi.nlm.nih.gov/Blast.cgi#alnHdr_237872438) ( FJ914712 ) | 99 | KJ542251 | Rc |
| 62 | Rct63 | *Penicillium halotolerans* ( NR111812 ) | 100 | KJ542252 | Rc |
| 63 | Rct64 | [*Aspergillus niger*](http://blast.ncbi.nlm.nih.gov/Blast.cgi#alnHdr_527851625) ( KF305758 ) | 100 | KJ542253 | Rc |
| 64 | Rct65 | *Aspergillus* *versicolor* ( KJ527011 ) | 100 | KJ542254 | Rc |
| 65 | Rct66 | [*Aspergillus versicolor*](http://blast.ncbi.nlm.nih.gov/Blast.cgi#alnHdr_459061852) ( KJ527011 ) | 100 | KJ542255 | Rc |
| 66 | Rct67 | [*Tetracladium sp.*](http://blast.ncbi.nlm.nih.gov/Blast.cgi#alnHdr_314947159) ( HQ747302 ) | 97 | KJ542256 | Rc |
| 67 | Rct68 | [*Botrytis elliptica*](http://blast.ncbi.nlm.nih.gov/Blast.cgi#alnHdr_373809852) ( AB693905 ) | 100 | KJ542257 | Rc |
| 68 | Rct69 | [*Coniochaeta* sp.](http://blast.ncbi.nlm.nih.gov/Blast.cgi#alnHdr_239948873) ( NR121473 ) | 98 | KJ542258 | Rc |
| 69 | Rct70 | [*Botryotinia fuckeliana*](http://blast.ncbi.nlm.nih.gov/Blast.cgi#alnHdr_540070776) ( KF532992 ) | 99 | KJ542259 | Rc |
| 70 | Rct71 | [*Neonectria ramulariae*](http://blast.ncbi.nlm.nih.gov/Blast.cgi#alnHdr_345447657) ( JF735314 ) | 99 | KJ542260 | Rc |
| 71 | Rac2 | *Phialocephala fortinii* ( KJ817297 ) | 99 | KJ542261 | Ra |
| 72 | Rac3 | *Phialocephala fortinii* ( KJ817297 ) | 99 | KJ542262 | Ra |
| 73 | Rac4 | *Cryptosporiopsis ericae* ( GU934585 ) | 100 | KJ542263 | Ra |
| 74 | Rac6 | *Phomopsis vaccinii* ( KC488259 ) | 99 | KJ542264 | Ra |
| 75 | Rac8 | *Aspergillus sydowii* ( KJ173528 ) | 100 | KJ542265 | Ra |
| 76 | Rac9 | [*Phomopsis conorum*](http://blast.ncbi.nlm.nih.gov/Blast.cgi#alnHdr_129562702)( DQ116553 ) | 99 | KJ542266 | Ra |
| 77 | Rac11 | [*Phoma herbarum*](http://blast.ncbi.nlm.nih.gov/Blast.cgi#alnHdr_459354696) ( KF986446 ) | 100 | KJ542267 | Ra |
| 78 | Rac12 | *Lachnum* sp. ( KJ529001 ) | 99 | KJ542268 | Ra |
| 79 | Rac14 | *Cryptosporiopsis ericae* ( AB369417 ) | 100 | KJ542269 | Ra |
| 80 | Rac15 | *Gibberella avenacea* ( FJ224099 ) | 99 | KJ542270 | Ra |
| 81 | Rac18 | *Umbelopsis* sp. ( JN198475 ) | 78 | KJ542271 | Ra |
| 82 | Rac25 | *Epicoccum nigrum* ( KF986447 ) | 100 | KJ542272 | Ra |
| 83 | Rac26 | [*Phialocephala fortinii*](http://blast.ncbi.nlm.nih.gov/Blast.cgi#alnHdr_217323322)( KJ817297 ) | 99 | KJ542273 | Ra |
| 84 | Rac27 | *Gibberella avenacea* ( FJ224099 ) | 100 | KJ542274 | Ra |
| 85 | Rac29 | *Epicoccum nigrum* ( KF986447 ) | 100 | KJ542275 | Ra |
| 86 | Rac30 | *Alternaria alternata* ( KJ716876 ) | 100 | KJ542276 | Ra |
| 87 | Rac32 | *Hypocrea longipilosa* ( JF303873 ) | 100 | KJ542277 | Ra |
| 88 | Rac36 | *Neonectria ramulariae* ( FR717232 ) | 100 | KJ542278 | Ra |

**Table S1.** Continued.

| **Code** | **Reference species (RS)** | **Closest species match (Accession No.)** | **% Identity** | **Accession No. of RS** | **Host** |
| --- | --- | --- | --- | --- | --- |
| 89 | Rac37 | [Sordariomycetes sp.](http://blast.ncbi.nlm.nih.gov/Blast.cgi#alnHdr_387351704)( GQ153034 ) | 99 | KJ542279 | Ra |
| 90 | Rac38 | *Cryptosporiopsis ericae* ( GU934585 ) | 100 | KJ542280 | Ra |
| 91 | Rac39 | *Penicillium thomii* ( JN624909 ) | 99 | KJ542281 | Ra |
| 92 | Rac40 | *Cryptosporiopsis ericae* ( GU934585 ) | 100 | KJ542282 | Ra |
| 93 | Rac41 | *Fusarium tricinctum* ( KJ598869 ) | 99 | KJ542283 | Ra |
| 94 | Rac43 | *Trichoderma harzianum* ( KM079608 ) | 100 | KJ542284 | Ra |
| 95 | Rac44 | *Phomopsis* sp. ( EF432292 ) | 99 | KJ542285 | Ra |
| 96 | Rac47 | *Phaeosphaeria* sp. ( GQ922523 ) | 96 | KJ542286 | Ra |
| 97 | Rac51 | [*Chaetomium angustispirale*](http://blast.ncbi.nlm.nih.gov/Blast.cgi#alnHdr_327532887)( JN209862 ) | 99 | KJ542287 | Ra |
| 98 | Rac52 | *Cladosporium cladosporioides* ( HQ443256 ) | 99 | KJ542288 | Ra |
| 99 | Rac53 | *Microsphaeropsis arundinis* ( JQ814844 ) | 99 | KJ542289 | Ra |
| 100 | Rac54 | *Phoma* sp. ( GU395514 ) | 99 | KJ542290 | Ra |
| 101 | Rac55 | *Phoma* sp. ( GU395514 ) | 99 | KJ542291 | Ra |
| 102 | Rac56 | *Phialocephala fortinii* ( AY394921 ) | 99 | KJ542292 | Ra |
| 103 | Rac57 | [*Phialocephala fortinii*](http://blast.ncbi.nlm.nih.gov/Blast.cgi#alnHdr_37624794) ( AY394921 ) | 99 | KJ542293 | Ra |
| 104 | Rac58 | *Alternaria alternata* ( KJ735924 ) | 100 | KJ542294 | Ra |
| 105 | Rac59 | *Phomopsis* sp. ( EF432292 ) | 99 | KJ542295 | Ra |
| 106 | Rac60 | *Phomopsis* sp. ( EF432292 ) | 99 | KJ542296 | Ra |
| 107 | Rac61 | *Phomopsis* sp. ( EF432292 ) | 99 | KJ542297 | Ra |
| 108 | Rac62 | *Aspergillus* *tennesseensis* ( KF986440 ) | 100 | KJ542298 | Ra |
| 109 | Rac63 | *Phialocephala fortinii* ( EU888625 ) | 100 | KJ542299 | Ra |
| 110 | Rac66 | *Phialocephala fortinii* ( EU888625 ) | 100 | KJ542300 | Ra |
| 111 | Rac69 | *Ceratobasidium* sp. ( AF354089 ) | 98 | KJ542301 | Ra |
| 112 | Rac71 | *Epicoccum nigrum* ( KF769446 ) | 100 | KJ542302 | Ra |
| 113 | Rac73 | *Cryptosporiopsis ericae* ( GU934585 ) | 100 | KJ542303 | Ra |
| 114 | Rac74 | *Cryptosporiopsis ericae* ( GU934585 ) | 100 | KJ542304 | Ra |
| 115 | Rac76 | *Lachnum* sp. ( FJ378862 ) | 99 | KJ542305 | Ra |
| 116 | Rac77 | *Aspergillus sydowii* ( KC795923 ) | 100 | KJ542306 | Ra |
| 117 | Rac79 | [*Aspergillus sydowii*](http://blast.ncbi.nlm.nih.gov/Blast.cgi#alnHdr_514996735) ( KC795923 ) | 100 | KJ542307 | Ra |
| 118 | Rac81 | [*Ceratobasidium* sp.](http://blast.ncbi.nlm.nih.gov/Blast.cgi#alnHdr_16565816) ( KC753438 ) | 99 | KJ542308 | Ra |
| 119 | Rac85 | [*Ceratobasidium* sp.](http://blast.ncbi.nlm.nih.gov/Blast.cgi#alnHdr_16565816)( KC753438 ) | 99 | KJ542309 | Ra |
| 120 | Rac88 | *Entrophospora* sp. ( HM208740 ) | 99 | KJ542310 | Ra |
| 121 | Rsc2 | *Epicoccum nigrum* ( JQ676202 ) | 99 | KJ542311 | Rs |
| 122 | Rsc3 | *Chaetomium globosum* ( KC603768 ) | 100 | KJ542312 | Rs |
| 123 | Rsc4 | *Trichoderma atroviride* ( JX045771 ) | 100 | KJ542313 | Rs |
| 124 | Rsc5 | *Biscogniauxia* sp. ( KF367566 ) | 96 | KJ542314 | Rs |
| 125 | Rsc7 | *Ilyonectria radicicola* ( KF895004 ) | 99 | KJ542315 | Rs |
| 126 | Rsc8 | *Fusarium sp.* ( AB587004 ) | 99 | KJ542316 | Rs |
| 127 | Rsc11 | [*Penicillium*](http://blast.ncbi.nlm.nih.gov/Blast.cgi#alnHdr_348161200) *lapidosum* ( NR121223 ) | 100 | KJ542317 | Rs |
| 128 | Rsc13 | *Lachnum* sp. ( KJ817276 ) | 99 | KJ542318 | Rs |
| 129 | Rsc15 | *Exophiala heteromorpha* ( AB190397 ) | 100 | KJ542319 | Rs |
| 130 | Rsc18 | [*Trimmatostroma* sp.](http://blast.ncbi.nlm.nih.gov/Blast.cgi#alnHdr_157277351) ( EU019299 ) | 95 | KJ542320 | Rs |
| 131 | Rsc20 | *Leptosphaeria* sp. ( JX401979 ) | 99 | KJ542321 | Rs |
| 132 | Rsc21 | *Leptosphaeria* sp. ( JX401979 ) | 99 | KJ542322 | Rs |

**Table S1.** Continued.

| **Code** | **Reference species (RS)** | **Closest species match (Accession No.)** | **% Identity** | **Accession No. of RS** | **Host** |
| --- | --- | --- | --- | --- | --- |
| 133 | Rsc22 | *Phialocephala fortinii* ( EU888625 ) | 100 | KJ542323 | Rs |
| 134 | Rsc23 | *Pseudaegerita viridis* ( EF029235 ) | 99 | KJ542324 | Rs |
| 135 | Rsc24 | Dothideomycetes sp. ( JQ759469 ) | 99 | KJ542325 | Rs |
| 136 | Rsc27 | *Leptosphaeria sp.* ( JX401979 ) | 99 | KJ542326 | Rs |
| 137 | Rsc29 | *Cytospora rhodophila* ( KF294010 ) | 99 | KJ542327 | Rs |
| 138 | Rsc31 | *Penicillium montanense* ( AF527058 ) | 100 | KJ542328 | Rs |
| 139 | Rsc33 | *Varicosporium elodeae* ( JX981463 ) | 99 | KJ542329 | Rs |
| 140 | Rsc36 | Leotiomycetes sp. ( KC007293 ) | 98 | KJ542330 | Rs |
| 141 | Rsc38 | *Cadophora melinii* ( HQ661098 ) | 99 | KJ542331 | Rs |
| 142 | Rsc39 | *Proliferodiscus alboviridis* ( U57990 ) | 98 | KJ542332 | Rs |
| 143 | Rsc40 | *Alternaria tenuissima* ( KJ728682 ) | 100 | KJ542333 | Rs |
| 144 | Rsc41 | *Alternaria alternata* ( KJ735924 ) | 100 | KJ542334 | Rs |
| 145 | Rsc42 | *Capronia* sp. ( AF050258 ) | 100 | KJ542335 | Rs |
| 146 | Rsc43 | *Phoma moricola* ( KF293794 ) | 99 | KJ542336 | Rs |
| 147 | Rsc44 | *Pestalotiopsis* sp. ( JN545787 ) | 95 | KJ542337 | Rs |
| 148 | Rsc45 | *Coprinellus xanthothrix* ( FJ755223 ) | 99 | KJ542338 | Rs |
| 149 | Rsc46 | *Leotiomycetes* sp. ( KC007293 ) | 99 | KJ542339 | Rs |
| 150 | Rsc47 | [*Leptosphaeria* sp.](http://blast.ncbi.nlm.nih.gov/Blast.cgi#alnHdr_406367517)( KC007293 ) | 99 | KJ542340 | Rs |
| 151 | Rsc49 | *Leptosphaeria* sp. ( KC007293 ) | 99 | KJ542341 | Rs |
| 152 | Rsc51 | *Rhizoctonia solani* ( KJ492168 ) | 100 | KJ542342 | Rs |
| 153 | Rsc52 | Leotiomycetes sp. ( KC007293 ) | 99 | KJ542343 | Rs |
| 154 | Rsc53 | Dothideomycetes sp. ( AB752255 ) | 99 | KJ542344 | Rs |
| 155 | Rsc55 | *Trimmatostroma betulinum* ( EU019299 ) | 95 | KJ542345 | Rs |
| 156 | Rsc57 | Dothideomycetes sp. ( AB752255 ) | 99 | KJ542346 | Rs |
| 157 | Rsc58 | *Gibberella avenacea* ( HQ020471 ) | 99 | KJ542347 | Rs |
| 158 | Rsc59 | *Alternaria* *alternata* ( KF990151 ) | 100 | KJ542348 | Rs |
| 159 | Rsc62 | *Phaeosphaeria* *nodorum* ( GQ922523 ) | 95 | KJ542349 | Rs |
| 160 | Rsc64 | *Leptosphaeria* sp. ( GU934536 ) | 99 | KJ542350 | Rs |
| 161 | Rsc65 | *Leptosphaeria* sp. ( GU934536 ) | 99 | KJ542351 | Rs |
| 162 | Rsc66 | [*Leptodontium* sp.](http://blast.ncbi.nlm.nih.gov/Blast.cgi#alnHdr_452108271)( KF428543 ) | 99 | KJ542352 | Rs |
| 163 | Rsc67 | [*Leptodontium* sp.](http://blast.ncbi.nlm.nih.gov/Blast.cgi#alnHdr_452108271)( KF428543 ) | 100 | KJ542353 | Rs |
| 164 | Rsc68 | *Leptodontium* sp. ( KF428543 ) | 100 | KJ542354 | Rs |
| 165 | Rsc70 | [*Leptodontium* sp.](http://blast.ncbi.nlm.nih.gov/Blast.cgi#alnHdr_452108271)( KF428543 ) | 100 | KJ542355 | Rs |
| 166 | Rsc71 | *Gibberella avenacea* ( FJ224099 ) | 100 | KJ542356 | Rs |
| 167 | Rsc72 | *Leptosphaeria* sp. ( GU934536 ) | 99 | KJ542357 | Rs |
| 168 | Rsc73 | *Leptosphaeria* sp. ( GU934536 ) | 99 | KJ542358 | Rs |

*Rc*, *Rhodiola crenulata;* *Ra, R. angusta* and *Rs, R. sachalinensis*; Rct: the isolates from *Rc* in Tibet; Rac and Rsc: the isolates from *Ra* and *Rs* in Changbai Mountain.
